# Microstructure and Mechanical Properties of Inverse Nanocomposite Made from Polylactide and Hydroxyapatite Nanoparticles

**DOI:** 10.3390/ma15010184

**Published:** 2021-12-27

**Authors:** Elżbieta Pietrzykowska, Barbara Romelczyk-Baishya, Agnieszka Chodara, Iwona Koltsov, Hilary Smogór, Jan Mizeracki, Zbigniew Pakieła, Witold Łojkowski

**Affiliations:** 1 Institute of High Pressure Physics, Polish Academy of Sciences, Sokolowska 29/37, 01-142 Warsaw, Poland; a.chodara@labnano.pl (A.C.); i.koltsov@labnano.pl (I.K.); j.mizeracki@labnano.pl (J.M.); w.lojkowski@labnano.pl (W.Ł.); 2 Faculty of Materials Science and Engineering, Warsaw University of Technology, Woloska 141, 02-507 Warsaw, Poland; barbara.baishya@pw.edu.pl (B.R.-B.); zbigniew.pakiela@pw.edu.pl (Z.P.); 3 NETZSCH Instrumenty, Halicka 9, 31-036 Krakow, Poland; chilary.smogor@netzsch.com

**Keywords:** inverse composites, biomaterials, mechanical properties

## Abstract

Polymer nanocomposites have been extensively researched for a variety of applications, including medical osteoregenerative implants. However, no satisfactory solution has yet been found for regeneration of big, and so-called critical, bone losses. The requirement is to create a resorbable material which is characterised by optimum porosity, sufficient strength, and elastic modulus matching that of the bone, thus stimulating tissue regrowth. Inverse nanocomposites, where the ceramic content is larger than the polymer content, are a recent development. Due to their high ceramic content, they may offer the required properties for bone implants, currently not met by polymer nanocomposites with a small number of nanoparticles. This paper presents inverse nanocomposites composed of bioresorbable nano crystalline hydroxyapatite (HAP NPs) and polylactide (PLLA), produced by cryomilling and a warm isostatic pressing method. The following compositions were studied: 25%, 50%, and 75% of HAP NPs by volume. The mechanical properties and structure of these composites were examined. It was discovered that 50% volume content was optimal as far as compressive strength and porosity are concerned. The inverse nanocomposite with 50% nanoceramics volume displayed a compressive strength of 99 ± 4 MPa, a contact angle of 50°, and 25% porosity, which make this material a candidate for further studies as a bioresorbable bone implant.

## 1. Introduction

Extensive research has been carried out on biomaterials that enable regeneration of critical bone loss by filling it with a bone-like material and on factors that stimulate bone tissue formation. The main difficulty in designing such solutions is to provide an implant with osteogenic properties combined with mechanical strength and a certain degradation profile [1,2,3,4,5,6,7]. Therefore, this article presents research on the microstructure and mechanical properties of a composite with a high proportion of HAP NPs and bioresorbable polylactide (PLLA), which is popularly used in orthopaedics. Changes occurring in the microstructure, in line with the increase in HAP NPs content, are described. Composites with a high content of ceramics differ from conventional materials in terms of their structure and properties and are called inversive composites. It is worth mentioning that hydroxyapatite also indicates a potential for differentiation of stem cells to osteoblasts, which is why it is such a promising material for orthopaedics [8,9].

Composites made of PLLA and bioactive ceramics, such as hydroxyapatite, are widely used in implantology for the purpose of improving clinical results. HAP NPs are added to matrices of orthopaedic implants based on polyesters, such as PLLA, in order to increase biocompatibility and Young’s modulus, but also to neutralize degraded and acidic monomers. 

In addition, HAP NPs are attractive due to the similarity of composition and particle size to the natural apatite present in bone tissue. The products typically encountered on the orthopedic implant market are mainly composites with a low HAP NPs content, up to 25%, characterized by relatively low mechanical properties but easily formable [10,11,12]. 

Nanocomposites are well known as materials where a small fraction of nanoparticles is added to improve their various properties. Due to the size in the nanometer range, a smaller fraction of a ceramic material is used, compared to conventional composites, and is usually in the range of a few percent [13,14]. Recently, much attention has been paid to inverse nanocomposites, where the polymer content is the same or even smaller than the ceramic content. In fact, the polymer is then filling the pores between ceramic nanoparticles. It might be expected that, with proper processing, such a structure would preserve the special properties of the nanoceramic while the polymer would see a decrease in the material brittleness, as well as more control of the material’s elastic modulus. Nanocomposites containing more than 25% by volume of nanofiller, i.e., 50% by weight, differ from conventional structures because the interactions adopted for polymer composites are modified with solid particles. They are called inverse materials [15,16].

Composites based on polyesters, such as polylactide (PLA), polycaprolactone (PCL), and their copolymers (PLGA, PLDLA, P(LLA-CL)) with bioactive particles of calcium phosphates, form a wide group of biomaterials [17,18]. They are produced most often by chemical methods (dissolving the polymer and adding calcium phosphate particles) as well as mechanical stirring, e.g., in extruders and spontaneous reactions of HAP NPs crystallization during the polymer synthesis. Composites with bioactive particles represent a promising solution to the well-known counter effects generally presented by conventional composites [19,20,21]. They are formed by injection, electrospinning, casting, cold or hot pressing, or 3D printing methods [22,23,24,25,26,27,28].

Bioresorbable composites based on polyesters are characterized by varied mechanical properties and biodegradation time, which result from differences in the physical properties of the polymer, such as molecular weight, polymerization degree, crystallinity, structure, additives, and stabilizing agents. A common problem related to the processing of composites based on polyesters is their decomposition, also known as thermal degradation or stability. For example, if PLLA is kept at a temperature above the melting temperature for a long time, its crystallinity and, consequently, its mechanical strength decreases [29,30,31,32,33,34,35,36,37,38,39,40,41,42].

Composites based on polyesters and bioactive ceramics, presented in the literature, can be divided into three groups depending on the quantity of calcium phosphates in the polymer matrix. Depending on this proportion of components, biomaterials differ in mechanical properties, biological activity and production method.

The first group contains composites with a small quantity of phosphate ceramics, such as HAP NPs, up to 20%. The assumption for these materials is to increase their biocompatibility in relation to pure polymers and to neutralize acidic products of their degradation. The literature also reports an increase in tensile strength and stiffness of the material by 10% [43]. Adding 5 wt% of bioactive particles is regarded as most beneficial in terms of mechanical properties, on condition that the composite has already achieved high dispersion and homogeneity [43,44,45,46,47,48]. This small quantity of bioactive particles increases the regeneration rate, as compared to a pure bioresorbable polymer, and helps the tissue overgrow the implant surface. Despite this, additional factors, such as bone morphogenetic protein (BMP), are required for these composites on the implant surface. Furthermore, 5% is regarded as insufficient for conversion of the material into natural tissue. Bonfield et al. demonstrate that the minimum volume percentage of HAP that is advantageous in terms of bone growth and conversion is ~20% [11]. 

The second group of these composites are those with the calcium phosphate content of up to 70% (by weight), i.e., a high volume proportion of ceramics (up to 50 vol%). The polymer fulfils the role of matrix in these composites. They are produced chemically or by extruders and are formed most often by the pressing or 3D printing methods. These composites achieve very good biological and mechanical properties [49,50,51,52]. However, disadvantageous degradation of such composites was reported in several papers. 

Three-dimensional printing applies a different approach to an orthopedic implant such as a porous scaffolding (porosity up to 40%, pore size above 200 μm), where blood and cells are able to penetrate and grow in. This material has a bigger surface of interaction with tissue and makes the regrowth of its whole volume possible. For these 3D printing materials, the authors concentrate on the biological properties and the regrowth mechanism. Mechanical properties in this case are described as sufficient, although the implant does not offer a load-bearing function. It must be secured with titanium plates which bear loads [48,49,50,51,52,53,54,55,56,57].

The third group are composites with an increased calcium phosphate content (above 50 vol%). For such composites, a high ceramic content leads to better mechanical properties compared to classical polymer composites, comparable to natural bone tissue. Rakovsky et al. describe PLLA-based composites with the content of bioactive ceramic particles ranging from 60 to 80 vol% with the use of high-pressure consolidation of 2.5 GPa at room temperature. They obtained dense nanocomposites with a porosity of approx. Moreover, 1% and compressive strength of 300 MPa for 60 vol% [53,54,55,56,57,58,59,60]. Pietrzykowska et al. obtained a composite with 80 wt% content of HAP NPs, characterized by a high mechanical strength. The obtained dense homogeneous composite is characterized by a porosity below 1% and the compressive strength of 374 MPa. That article presents optimization of forming in terms of mechanical properties and achievement of a material with a strength comparable to natural bone. This composite is characterised by a high homogeneity on the nanometric level [61]. Jakus et al. describe a flexible composite with the calcium phosphate content of 90% for 3D printing, the properties of which are controlled by the applied polymer solvents. In their paper, they present an interesting microstructure of big hydroxyapatite granules surrounded by a polymer, which resembles a pomegranate fruit [62]. These two publications present materials with different microstructures, homogeneity, and mechanical properties. There is no information about the impact of the ceramic particles on the polymer matrix and its thermal stability. Therefore, the present paper discusses the diversity of microstructures and their impact on the final properties of the composite.

The composite microstructure is influenced by numerous factors. The type of the used hydroxyapatite, its size and shape are of fundamental importance. Nanometric particles with the spherical shape are regarded as most favourable [59]. As far as the methods of obtaining composites are concerned, chemical methods and extrusion at an increased temperature make it possible to obtain most homogeneous composites, which is proved by Mathieu et al. They indicate also that a material after hot extrusion is characterised by a higher tensile strength and a higher tensile elongation at break. On the other hand, mechanical milling and chemical methods allow a higher content of ceramic particles, which still requires expensive processing for several hours. In the case of mechanical mixing, the ceramic particles in the microstructure are often located near the places where polymer granules contact one another [48,53,59]. 

The method of forming influences in particular the porosity. In the case of isostatic pressing, the higher the pressure, time, or temperature, the lower the porosity [56]. 

The mechanical properties result from the microstructure, the composition, the connection between the components, and the porosity. It is also common knowledge that the mechanical strength of a material decreases in line with the increase in the porosity.

Changes occurring in the matrix, i.e., in the polymer, as a result of the polymers formed, exert a considerable influence on the final properties of the composite, its stability, repeatability, and degradation time. Literature describes consequences of these changes as a decrease in glass transition and melting temperatures and crystallinity. A short composite degradation time in the in vitro conditions is also reported. Numerous publications provide that degradation is more marked in composites with a higher ceramic content due to the lower quantity of polymer between ceramic particles. However, the aspect of thermal stability and changes occurring in the polymer depending on the proportion of the ceramic particles does not provide information about a possibility to obtain a high-strength and bioactive composite based on a bioresorbable polymer [63,64,65,66,67].

In designing biomaterials, one should also take into consideration their stability during sterilisation and methods of modification, e.g., ionizing radiation [68,69].

The present paper describes fabrication method, the microstructure and mechanical properties of inverse nanocomposites with the matrix of bioresorbable PLLA with varied HAP NPs contents. A strong emphasis is put on the characterization of polymer changes during the forming and on the changes occurring in the polymer related to the increase in the quantity of HAP NPs. Changes in the contact angle, the bending strength and crystallinity of composites, melting temperature, and glass transition temperature of the polymer are examined. The aim of this study is to reveal the changes occurring in the microstructure of inverse composites, which help to characterize their properties and select the optimum content.

## 2. Materials and Methods

### 2.1. Materials

The composite was produced of bioactive nanoparticles of hydroxyapatite (HAP NPs) and a bioresorbable polyester: polylactide (PLLA).

**Figure 1:**
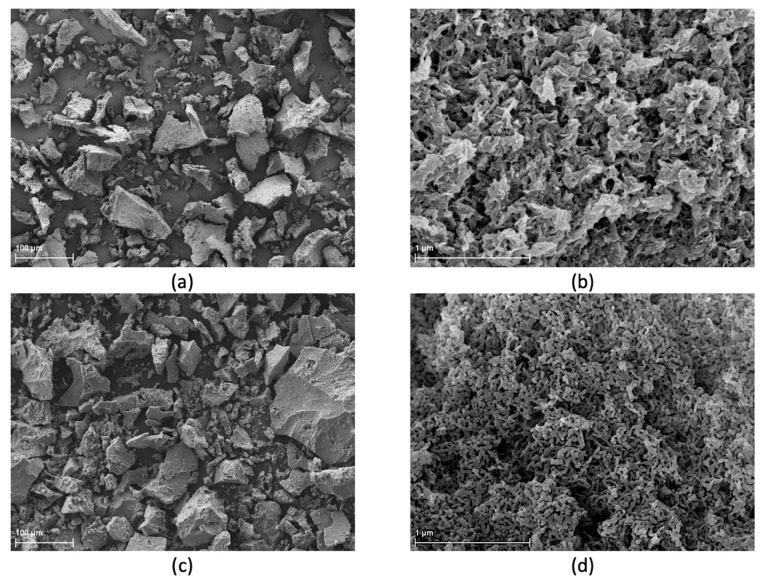
SEM micrographs of powder (**a**,**b**) raw HAP NPs, (**c**,**d**) heated HAP NPs.

**Figure 2:**
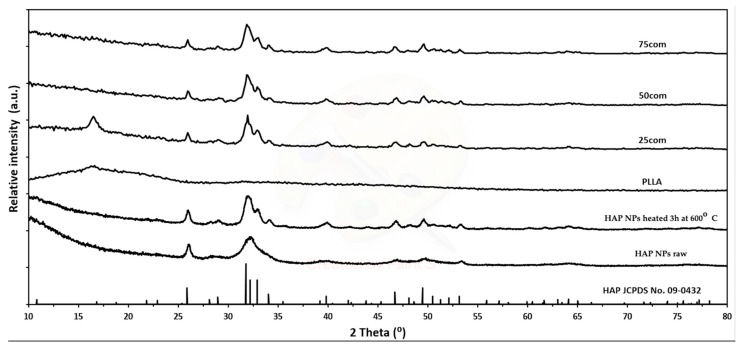
XRD of the HAP NPs (raw), HAP NPs (heated at 600 °C) powder and of the composite after warm isostatic pressing at 170 °C.

HAP NPs (raw) were synthesized at the Institute of High Pressure Physics of the Polish Academy of Sciences by the method described in the publication by Kuśnieruk et al. [70]. They are a crystalline powder with the density of 2.86 g/cm^3^ and the specific surface area of 260 m^2^/g. The average particle size calculated based on the specific surface area and the skeletal density is 8 nm. HAP NPs are nonstoichiometric and have the structure of needles, with a Ca–P ratio is 1.65. Figure 1 shows X-ray diffraction (XRD) measurements of the HAP NPs, which reveal that this material is pure in terms of phase. Figure 2 presents the agglomerate size, which is 100 μm (Figure 1a) and the particle size (Figure 1b) for HAP NPs.

PLLA (Sigma-Aldrich, Buchs, Switzerland), i.e., a bioresorbable thermoplastic polymer, in the form of granulate with the viscosity of −4.0 dL/g 0.1 (*w*/*v*) and the density of 1.24 g/cm^3^, was used in the tests.

### 2.2. Methods

The HAP NPs (raw) powder was heated at 600 °C to remove the adsorbed water. According to Pietrzykowska et al., this is an effective method of preparing a nanopowder for application in composites obtained by cryomilling and subjected to isostatic pressing at an increased temperature [71]. The HAP NPs powder was placed in the furnace and dried at the temperature of 600 °C for 3 h, with the heating increase rate of 2 °C/min. After soaking at 600 °C, it was cooled down inside the furnace until it reached the room temperature. The obtained powder will be designated as HAP NPs. 

Then samples were fabricated using a two stage method. First, a mixture of polymer particles and nanopowder was milled at liquid nitrogen temperature (criomilling), leading to the formation of composite granules, and isostatic pressing of the granules at pressure (Warm Isostatic Pressing). The method will be further called CMWIP.

At the first stage, a composite granulate was prepared with varied contents of bioactive particles: 25, 50, and 75 vol% of HAP NPs, which are designated as 25com, 50com, and 75com, respectively.

The 6775 Freezer/Mill® Cryogenic Grinder (SPEX SamplePrep, Metuchen, NJ, USA) was used for obtaining the composite granulate. After weighing the HAP NPs powder, PLLA was placed in a steel tube together with a steel spindle. Subsequently, the tube was fastened in the mill, whose tub was filled with liquid nitrogen. The tube was precooled for 15 min. After cooling down, the materials were milled in 3 cycles for 2 min, with cooling between cycles for 5 min.

The composites were dried at the temperature of 50 °C and in a vacuum of 260 mbar for 48 h. Subsequently, the composites were formed in the conditions of warm isostatic pressing at the temperature of 170 °C and the pressure of 65 MPa. 

The composite granulate was placed in a flexible mold and subjected to isostatic pressing in a steel chamber filled with oil heated to 170 °C. The samples were kept at the temperature of 170 °C for 15 min and, afterwards, subjected to isostatic pressing at the temperature of 170 °C and the pressure of 65 MPa for 12 min. Five samples, weighing 1–2 g each (depending on composite), were prepared. Their shape was rectangular cuboid sized 4 × 4 × 35 mm.

A hydraulic chamber and an isostatic press (model LCP20, Institute of High Pressure PAS, Warsaw, Poland) were used for the forming. The temperature was given and controlled by a proportional–integral–derivative controller (model RE93, Lumel S.A., Zielona Góra, Poland), together with a temperature sensor (T type thermocouple, model T-208p-K-1-300-40-2,5-SO-1-6-400, Termo-Precyzja, Wrocław, Poland).

The density measurements were performed with a helium pycnometer (model: AccuPyc 1330, Micromeritics, Norcross, GA, USA) in compliance with ISO 12154:2014 procedure at a temperature of 25 ± 2 °C.

Each sample was rinsed 50 times in helium and, subsequently, 50 measurements were performed. The results of this were then averaged. 

The specific surface area (SSA) of the powders was measured by the Brunauer-Emmett-Teller (BET) method (model: Gemini 2360, V 2.01, Micromeritics, Norcross, GA, USA) in compliance with ISO 9277:2010 procedure. The average diameter of the particles was calculated based on the specific surface area and density, assuming that all of the particles were spherical and identical [72].

Thermal stability of samples were investigated using Differential Scanning Calorimetry (DSC), method. The experiments were carried out on Netzsch DSC 214 Polyma (Netzsch-Gerätebau GmbH, Selb, Germany) mechanical cooling system (intracooler IC70, Netzsch-Gerätebau GmbH, Selb, Germany, with min. temp. −75C) was used for DSC furnace cooling. The tests were performed in aluminum cancavus crucibles where approximately 15 mg of samples were used. The experiments were designed with a heating rate of 10° K/min under a constant flow of nitrogen (25 mL/min). The experiment involved 3 cycles: 1st heating, cooling, followed by 2nd heating. Each heating cycle took place from approximately −20 °C up to 225 °C, while each cooling cycle took place from 225 °C to −20 °C.

The phase composition of the reaction products was analyzed by powder X-ray diffraction (Panalytical X’Pert PRO diractometer, Cu K↵1, Panalytical, Almelo, The Netherlands). The patterns were collected at room temperature in the two-theta range of 10–100 and with a step of 0.03. 

SEM: The materials’ structure was examined by scanning electron microscopy (SEM) using the Ultra Plus microscope (ZEISS, Oberkochen, Germany). The uniformity of phase distribution was studied by means of EDS with the use of the EDS Bruker mod. Quantax 400 connected to the SEM Zeiss Ultra Plus with.

Before the measurement, the samples were coated with a nanometric gold layer.

Three-point bending tests were carried out at room temperature using an QTest/10 (MTS, Eden Prairie, MN, USA) universal testing machine equipped with a ± 10 kN transducer. The deflection was measured by an MTS extensometer. The tests were conducted under the displacement control mode. The crosshead velocity was 0.5 mm/min. The minimum three samples with the length of 20 mm and the cross-section of 4 mm × 4 mm were used for each test. The span was 12 mm. Based on the stress-strain curve, the Young’s modulus (E), ultimate bending strength (UBS) and deflection were calculated. 

Uniaxial compression tests were carried out at room temperature using a Zwick 005 universal testing machine. Tests were conducted under the displacement control mode with the crosshead velocity of 20 μm/min. Samples sized 6 mm in height and 3 mm × 3 mm in cross-section were used for those tests. Three samples were measured for each material. Based on the load displacement data, the ultimate compression strength (UCS) and strain were estimated.

Contact angles indicating the wetting ability of the materials were measured by the drop shape analysis (DSA 100, KRüSS, Hamburg, Germany) at room temperature. Then, 3 μL of deionized water was dropped on the sample surfaces. At least five measurements were performed at different locations and the results were averaged. The samples were polished with sandpaper before measurement. They were then dried for 24 h in a vacuum dryer at the temperature of 50 °C under the pressure of 400 mBar.

## 3. Results

### 3.1. HAP NPs Powder Optimisation

The optimization procedure was intended to increase the nanoparticles size and reduce their water content. Figure 1a,b present the size of agglomerates and the sizes and shapes of particles of pure HAP NPs powders. The changes that occurred after the heating process at 600 °C are presented in Figure 1c,d. Heated HAP NPs have the form of spherical particles, which results from their growth. In addition, a decrease in the powder specific surface area from 260 m^2^/g to 72 m^2^/g and an increase in the density to 3.0 g/cm^3^ were observed, in agreement with an increase in grain size to 29 nm (Table 1). 

However, changes in the size of particles and agglomerates can be considered as small and insignificant. XRD (Figure 2) and SEM (Figure 1b) tests confirmed the preservation of the nanometric structure and above all the phase purity of the HAP NPs despite the increase in density. An XRD analysis of all HAP NPs and composites powders obtained was carried out. The diffraction patterns showed peaks from hydroxyapatite, indicating that it was a crystalline and phase pure material. The small increase in the width peaks for HAP NPs, as compared to HAP NPs, corresponds to a slight increase in hydroxyapatite grain size. For the PLLA and 25com samples, a peak appeared at account 16.4, i.e., from the polymer. For 25com, a peak was present but weakly undersized. The sizes of these peaks related to the degree of crystallinity of the polymer. The peak was not observed for the 50com and 75com composites, due to the high volume amount of nanoparticles in the polymer matrix. 

### 3.2. Composite Granulate 

**Figure 3:**
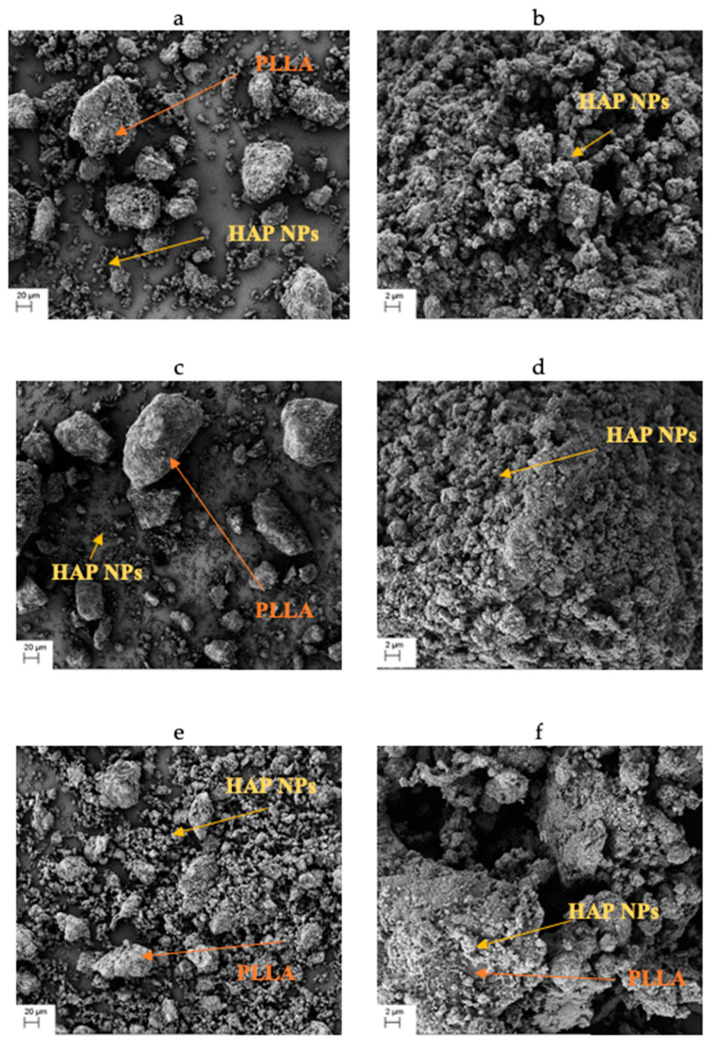
Composite granules for various proportions of HAP NPs, 25com (**a**,**b**), 50com (**c**,**d**), 75com (**e**,**f**).

The obtained composite granulate after cryomilling has an irregular shape. Both HAP’ NPs agglomerates and PLLA granules are visible. It can be observed in the SEM images in Figure 3 that the increased quantity of HAP NPs is accompanied by a greater fragmentation and a smaller size of the PLLA granules, which contributes positively to the composite homogeneity. The 25com composite granulate (Figure 3a) is several times the size of the 75com granulate (Figure 3e). Moreover, HAP NPs aggregates are visible on the PLLA granule surface in all composites (Figure 3b,d,f) and a single aggregate has the size below 10 μm. PLLA granules were crushed below 100 μm and HAP NPs with a form of 10-μm aggregates. 

The obtained granulates differ in density and specific surface area. Granulate density and specific surface area increase in line with the increase in the quantity of HAP NPs in the composite. The results and composition for composite granules are presented in Table 1. With an increase in the amount of HAP NPs and a decrease in the fragmentation, the specific surface area of the composite granulate increases.

HAP NPs powder has a specific surface area of 72 m^2^/g. For granules, it ranges from 0.5 to 53 m^2^/g.

After the warm isostatic pressing, solid samples were obtained, which were subjected to observations of microstructure and tests of physical properties.

### 3.3. Composite Microstructure

**Figure 4:**
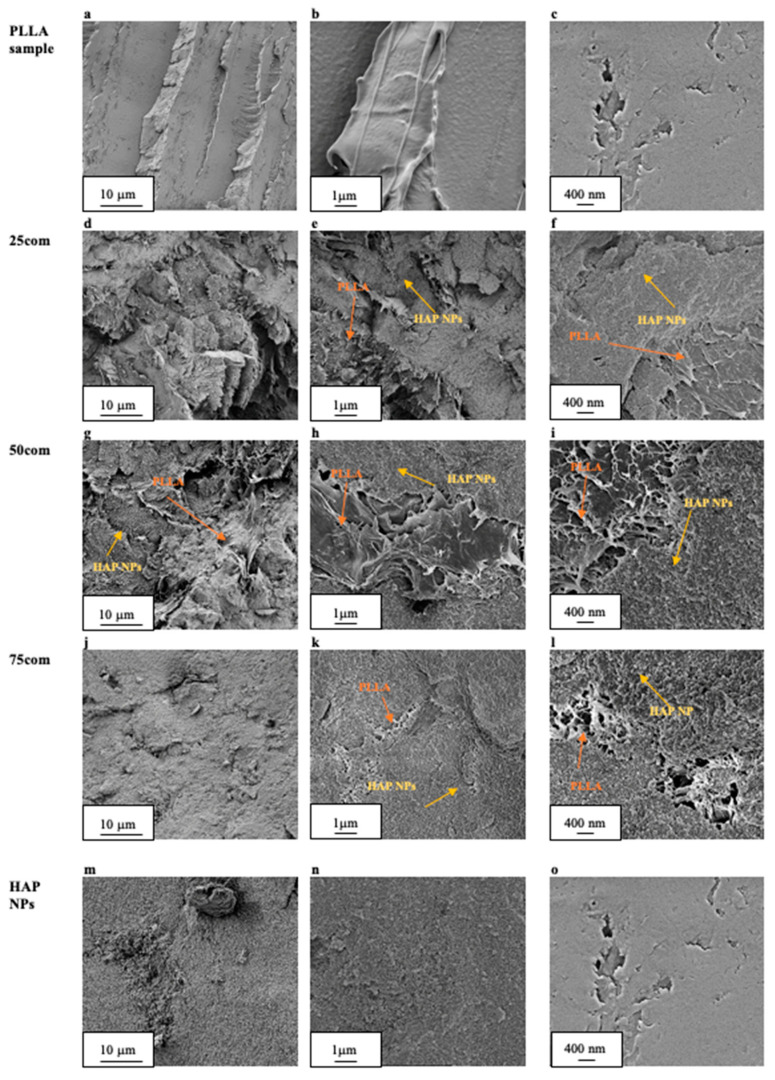
Fractures of samples and composites after the three-point bending test; PLLA (**a**–**c**), 25com (**d**–**f**), 50com (**g**–**j**), 75com (**j**–**l**) and HAP NPs (**m**–**o**).

The composite microstructure changes depending on the composition, i.e., in our case, we observed changes because of the proportion of PLLA to HAP NPs. The volume content for the produced inverse nanocomposite is 25, 50, and 75%, respectively, and changes were observed on sample fractures after the warm isostatic pressing, which is presented in Figure 4d,l. Figure 4a,c present the fracture of PLLA sample, with different magnifications. As can be observed, the PLLA surface is smooth but brittle.

The fracture of the 25com (Figure 4d), as compared with the PLLA sample, is also brittle but more developed. On the surface of 25com, HAP NPs (Figure 4e,f) are observed as aggregates with a size below 10 μm (Figure 4d). It can be inferred based on Figure 4f that there is a connection between the matrix and the ceramic particles in the form of polymer bridges. Furthermore, 25com contains areas where pieces of polymer in the form of threads are visible (Figure 4e). This difference results from the addition of HAP NPs as a component and from changes caused during the forming. This is the reason for the thread-like PLLA structures in Figure 4e,f. The material is continuous, and single pores are observable (porosity 3%). 

In the obtained microstructures of the 50com composite, the proportions of the ceramic particles and PLLA by volume are equal. The fracture of 50com has the form of flakes (Figure 4g). The microstructure is infiltrated with the polymer. Just like in the case of 25com, places with pieces of the polymer, sized 2 μm, are visible. Figure 4g discloses HAP NP areas surrounded by PLLA “moustache”, which forms a connection with HAP NPs as bridges (Figure 4i). What can be observed is a porosity of the composite, regular pores, and spots with an irregular and looser thread-like PLLA structure (Figure 4i). The greater magnifications of the fracture of 50com reveal that the structure is well mixed, while polymer present in the composite not only surrounds the ceramic HAP NPs but also forms polymer islands. The micron pieces of PLLA and the infiltration of 50com demonstrate the effect of the method of formation under pressure at an increased temperature. 

**Figure 5:**
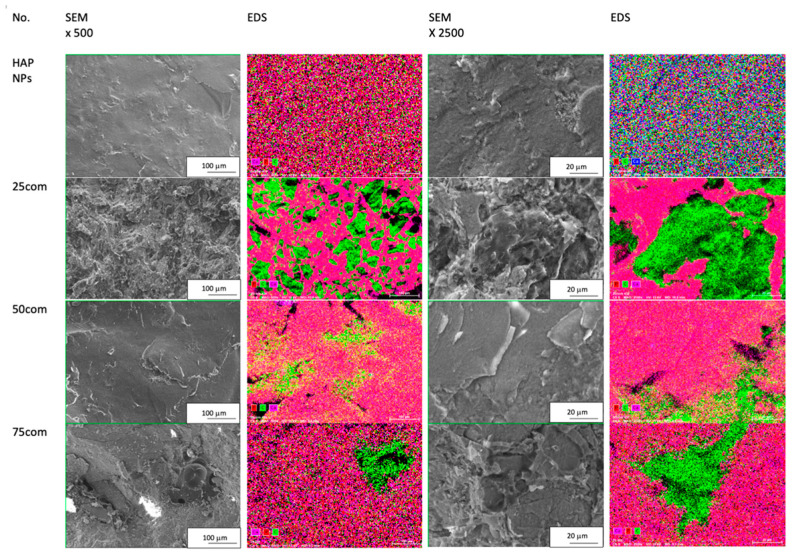
Collection of SEM images; EDS mapping for HAP NPs sample and 25com, 50com, and 75com composites.

The fracture of 75com is more similar to the HAP NPs sample (Figure 4m,o), while the HAP NPs are clearly visible. Greater magnifications (Figure 4l) reveal PLLA islands in the 75com composite similarly to the 50com composite, but they are considerably smaller. Furthermore, micrometric pores were observed in 75com (Figure 4j), which results from the greater difficulty in forming a composite with such a high proportion of ceramics. The fracture of 75com is brittle, not much developed, and is clearly distinct from that present in the 25com and 50com composites. The phase distribution and homogeneity were confirmed by EDS mapping shown in Figure 5. The EDS mapping was performed for the fracture surfaces of the surface of the 25com, 50com, 75com, and HAP NP samples. In the case of the 25com sample, a mosaic is visible, where PLLA areas can be distinguished easily from HAP NP areas. The distribution of HAP NPs and PLLA is uniform. A considerable increase in areas with HAP NPs is seen in comparison to the 25com. There are 100-μm-diameter areas of PLLA where Ca and P signals are visible, thus proving that HAP NPs aggregates are present there. 

### 3.4. Contact Angle

When analyzing the contact angle for the composites, a clear decrease in hydrophobicity of the composite in line with the increase in the HAP NPs quantity is observable. The contact angle for the PLLA sample is 70°, while it is 24°for the HAP NPs sample. The results of the composites fall between these values, proportional to the bioactive ceramic content. The results are provided in Table 2. Attention should be drawn to the quantity of the HAP NPs present on the material surface. The higher the content and dispersion, the greater the effect of HAP NPs on the contact angle.

When a drop of water was placed on the surface of a HAP NPs pastille, it was adsorbed. This is caused by the high quantity of nanopores in the HAP NPs sample, which is why the contact angle was measured 30 s after the drop was placed on the pastille surface for all samples. Calcium phosphates, including HAP NPs, are a hydrophilic material due to the hydroxyl groups on their structure. 

When the water drop was placed on the PLLA sample, it remained on its surface with the preserved shape. The size and shape of the drop proves the hydrophobic properties of PLLA. 

In the composites, the drop changes in line with the change in the HAP NPs quantity on the sample surface. For 25com, a slight flattening of the drop is observable. This effect is considerably more visible for the 50com and 75com samples. The drop on the 75com surface looks similar to HAP NPs, which confirms the high dispersion of the composite. 

**Figure 6:**
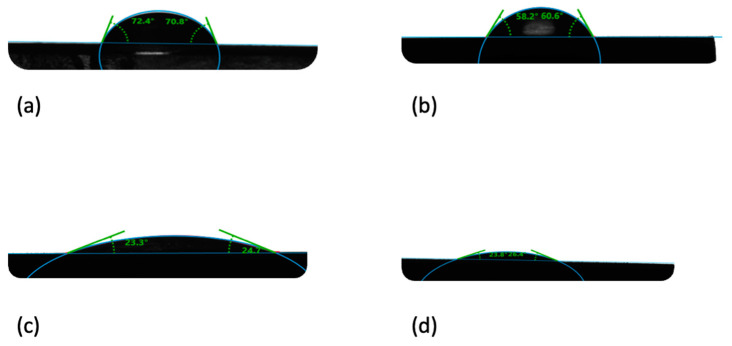
Contact angle of composites: (**a**) PLLA sample, (**b**) 25com, (**c**) 75com, (**d**) HAP NPs sample.

Figure 6 presents changes in “flattening” the drop depending on the composite, as compared to pure PLLA and HAP NPs samples. 

### 3.5. Mechanical Properties 

The mechanical properties of the 25com, 50com, and 75com composites were estimated and compared with pure PLLA and HAP NPs samples obtained, according to the same procedure. Bending strength, strain and Young’s modulus, and compressive strength were determined. The average values of the parameters mentioned above are presented in Table 3.

The bending strength of pure PLLA sample is 61 ± 6 MPa and decreases in line with the increasing content of HAP NPs. When comparing the bending strength of the pure HAP NPs sample and of the composite, their strengthening is observed. A pure HAP NPs sample achieved the strength of 11 ± 3 MPa. The bending strength values for 25com, 50com, and 75com are 28 ± 6 MPa, 19 ± 3 MPa, and 14 ± 2 MPa, respectively. Attention should be paid to the high porosity of those composites, as it is known that the porosity decreases the mechanical strength. It is an interesting result that 50com has the highest porosity (25%) and has comparable mechanical properties with 25com, which obtained 5% porosity. An addition of HAP NPs results in a drop of deflection for bend testing. Even a small addition of HAP results in a substantial decrease in strain. For the PLLA sample, the strain of 4.01 ± 0.43 was calculated, while for composites it was less than 1%. During the bending test for composite samples, the crack propagation was easier because of the presence of brittle HAP NPs. 

The compressive strength for all composites is higher than for pure PLLA and HAP NPs samples. The highest compressive strength observed for 25com is 114 ± 2 MPa. For 50com, it is 99 ± 4 MPa. The porosity for HAP NPs sample is 35% and the compression strength is 24 ± 4MPa. Despite a low porosity (18%), 75com has the compressive strength of 78 ± 7 MPa, which indicates that the material is more brittle and looks similar to HAP NPs sample. During the compression, a decrease in strain value in line with an increase in HAP NPs content was observed. This was caused by the brittleness of HAP NPs but the reduction is less significant than for the bending test. For compression, both pores and brittle particles of HAP exert a smaller influence on the crack path.

The Young’s modulus of the obtained composites increases in line with the HAP NPs proportion. The value of Young’s modulus for pure PLLA sample is 1603 ± 175 MPa, while for 25com composites, this value increases considerably to 3289 ± 497 MPa. It may be presumed that the HAP NPs with a big specific surface area proposed in this paper and the methods of obtaining the composite led to the strengthening of the role of the surface of HAP NPs. The field of contact between the filler (HAP NPs) and the matrix (PLLA) increases the wetting ability of HAP NPs with PLLA, thereby initiating the effect of polymer matrix strengthening by increasing the strength of its bond with the filler. This effect is stronger for 50com and 75com, thanks to which the Young’s modulus of the composites is 3619 ± 295 MPa and 7317 ± 197 MPa, respectively.

### 3.6. DSC Results 

DSC tests were carried out to determine the impact of HAP NPs on PLLA thermal properties, including crystallinity degree. In order to control the mechanical properties and the polymer degradation, an attempt was made to describe the thermal properties of the composite arising from selection of an appropriate amount of HAP NPs. 

All DSC curves show a similar trend. The shifts of the peaks from the appearing transition temperatures and their intensity are a result of the composites’ chemical composition, which stems from the structural changes in the amorphous–crystalline fraction in the composite.

**Figure 7:**
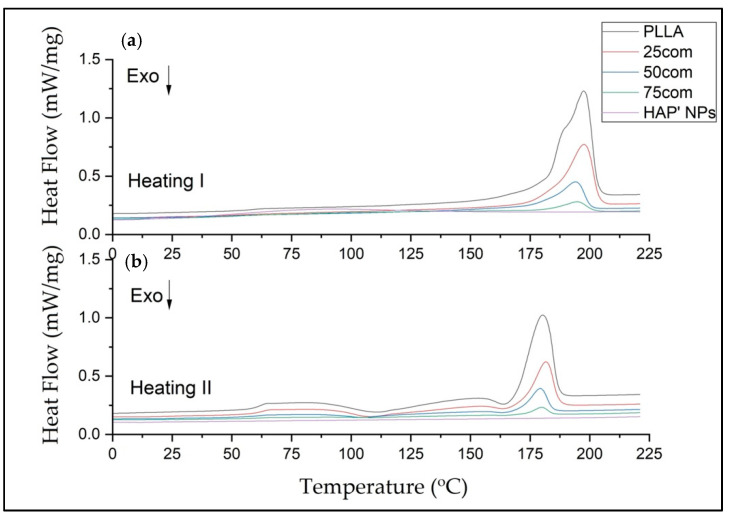
DSC thermograms of composites 25com, 50com, and 75com, as well as HAP NPs and PLLA samples, after first and second heating (a and b, respectively).

In our study, the DSC was evaluated in three steps: heating (first heating), cooling, and second heating for warm isostatic pressed PLLA and HAP NPs samples and composites. Figure 7 presents DSC curves for the 25com, 50com, and 75com composites, as well as HAP NP and PLLA samples, after the first and second heating. The DSC curve of the pure PLLA sample shows a glass transition temperature (Tg), cold crystallization temperatures (Tc), melting temperature (Tm), crystallization, and melting enthalpies. The DSC signals of the 25com, 50com, and 75com composites are similar. The curve for the HAP NPs sample shows changes associated with evaporation of water from the particle surface, visible only in the first heating but no characteristic temperatures in the second heating. All changes recorded in the composites originate from PLLA phase, because the HAP NPs in the temperature range studied are stable.

Table 4 presents the numerical values of Tg, Tc, Tm, Δ*Hc*, and Δ*Hm*, as well as the crystallinity degree (Xc’) for the PLLA sample and the composites.

Xc’ is the degree of the PLLA’s crystallinity, which can be calculated from Equation (1) according to the article [43], taking into account the mass fraction (*W*) of the polymer in the whole composite sample mas, as follows

(1) $$\mathrm{Xc}\prime =\frac{\Delta H}{\Delta H\begin{array}{c} 0\\ m \end{array}\times W}\times 100$$

where Δ*H* = Δ*H_m_* − Δ*Hc* represents the data from the heating cycle, and Δ*Hc* is the cold crystallization enthalpy. Δ$H\begin{array}{c} 0\\ m \end{array}$ is the melting enthalpy of 100% crystalline polymer, which was taken to be 93.6 J/g for PLLA.

The Tg of pure PLLA sample is 57.50 °C in the first heating and increases to 60.7 °C for the second heating. Generally, Tg increases between the first and the second heating for all samples. The difference between the first and the second heating for pure PLLA sample is 3.2 °C and, for the composites, it is much greater, up to 10° C, which means that HAP NPs change the mobility of polymer chains (after cold crystallization). For composites, Tg from the first heating is lower than in pure PLLA sample. This effect is related to the presence of water on the surface of HAP NPa. 

The Tc results are 110.7 and 163.5 °C for the PLLA sample. The first Tc is a little bit lower for the composites, i.e., 104 °C up to 108 °C, so crystallization likely starts more easily in the composites. The difference for the second Tc is not greater than 1 degree, so this value is negligible. 

**Figure 8:**
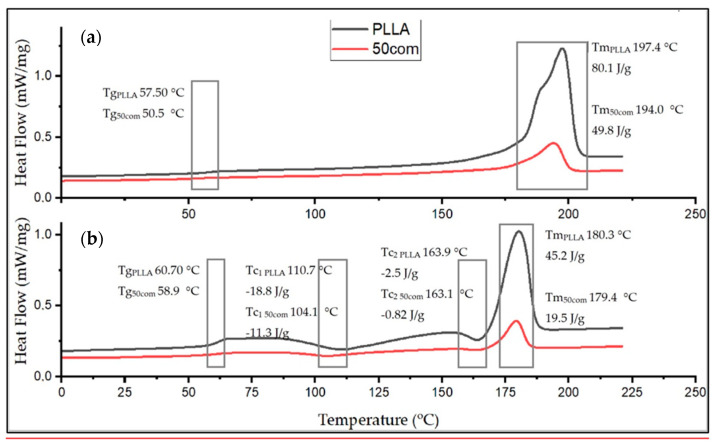
DSC thermograms of PLLA sample and 50com, after first and second heating ((**a,b**), respectively).

The Tm drops between the first and the second heating for both PLLA sample and the composites. The 25com composite has a Tm close to the pure PLLA sample. For a composite with a high content of HAP NPs, i.e., 50com and 75com, the melting temperature (Tm) drops to 194 °C during the first heating (for pure PLLA sample it is 197.4 °C). Tm after second heating is almost the same for all samples. Figure 8 shows the comparison of the curves for PLLA sample and 50com samples after the first and second heating. The temperature shifts of Tg, Tc, and Tm, as well as the enthalpy value, change. Changes in the area under the Tm and Tc peaks can be observed; as the amount of HAP NPs increases, the enthalpies of melting and crystallization decrease. 

Xc for the PLLA sample is 91.4%, while it is 67.8% for 25com, 49.8% for 50com, abd approx. 29.8% for 75com (during the first heating). The Xc after the first heating corresponds to the state in which the material was after the warm isostatic pressing. After the second heating, the decrease in the degree of crystallinity is greater. As the number of HAP NPs increases, the crystallinity of PLLA phase in composites decreases.

**Figure 9:**
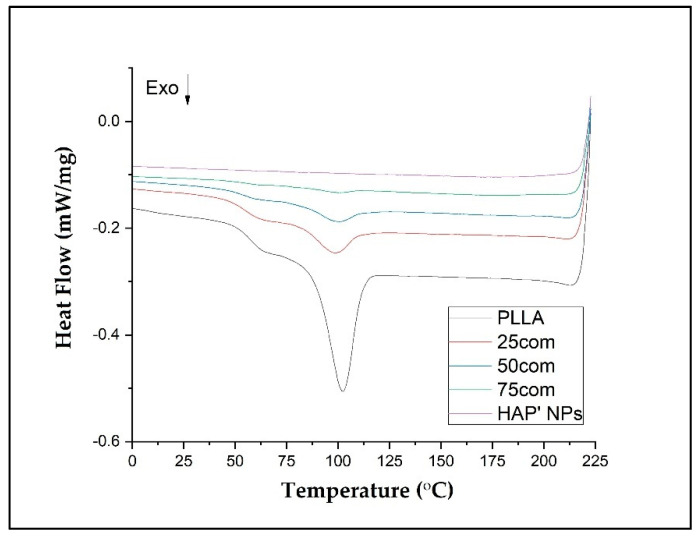
DSC curves of cooling segments for composites 25com, 50com, and 75com, as well as HAP NPs and PLLA samples.

Figure 9 shows the crystallization of the samples (PLLA, 25com, 50com, and 75com) during cooling (after the first heating). The crystallization temperature (Tcc) for the PLLA sample is 102.4 °C, while for composites it is lower (about 2 °C). In addition, the surface areas under the Tcc peaks decrease with increasing HAP NPs. That is, as HAP NPs increase, fewer crystals are formed in the polylactide present in the composite. HAP NPs makes crystallization more difficult.

## 4. Discussion

The aim of this study was to understand the impact of hydroxyapatite nanoparticles on the structure and properties of nanocomposites. The authors present the patented method CMWIP for the preparation of inverse nanocomposites by cryomilling and warm isostatic pressing. Thanks to this method, nanocomposites with high nanoparticle content can be produced. Therefore, the opportunity is open to fine-tune the properties of the composites and correlate them with structure changes. Special attention was given to the thermal properties of the PLLA phase as a function of composition in the composites (25com, 50com, and 75com).

The literature contains many examples which confirm the biological properties of HAP–PLLA composites, with first publications dating back to 2001. However, such studies do not provide a solution of a bioresorbable implant that, at the same time, carries the stresses. Composites characterized by compressive strength similar to bone tissue are still being sought. Bioresorbable orthopedic implants available on the market do not reach 100 MPa and require strengthening with, e.g., a titanium rail after the implantation. Although the literature presents composites characterized by considerably higher mechanical properties, their porosity, which is regarded as necessary for the bone tissue to overgrow the implant, is negligible: 1–10%. Therefore, we believe that the inverse composites presented by us are interesting and constitute an important achievement for the development of orthopedic implants.

The main component of described inverse nanocomposite is HAP NPs particles with a grain size of 8 nm. The authors found no other work with HAP NPs with such a low grain size. A technological challenge was to reduce the water content in the produced composites, i.e., which mostly come from the surface of HAP NPs. Szałaj et al. [73] have shown that nanometric hydroxyapatite particles used in the study strongly absorb water from the ambient atmosphere. As proved by Kuśnieruk et al., HAP NPs contain approx. 10 wt% of water on their surface and in their structure, proved by thermogravimetric studies. During heating of HAP NPs at 200 °C, 5.85 wt% of water is released [70]. Following our heating at 600 °C, the quantity of the released water decreases to 1.54 wt%, which is an acceptable value for composites with PLLA. The development of the specific surface area is due to the use of the patented HAP NPs, while the final value of the grain size is 29 nm. It is known from the literature that PLLA should be dried to below 0.025% for processing [22]. The knowledge of how to control the HAP NPs surface is very essential for composites with PLLA.

The used CAWIP contributes to the unique structure of our inverse nanocomposites and does not require the application of chemical solvents, applied in other methods, which are toxic to the organism and the environment. Obtained composite granules made by cryomilling were used to produce the inverse nanocomposite. The resulting granules exhibited sufficient homogeneity to produce inverse nanocomposites. The particle size distribution was below 100 μm. 

Isostatic pressing permits to create samples of various shapes, such as a wedge, and can help to thread or to create an item with an opening. Increasing the temperature (up to 170 °C) reduces the viscosity of the polymer component and permits to increase the density of the produced body [61]. By varying the consolidation pressure, time, and temperature, it is possible to control the porosity of these materials. These parameters need to be adjusted to the composition of the samples. 

**Figure 10:**
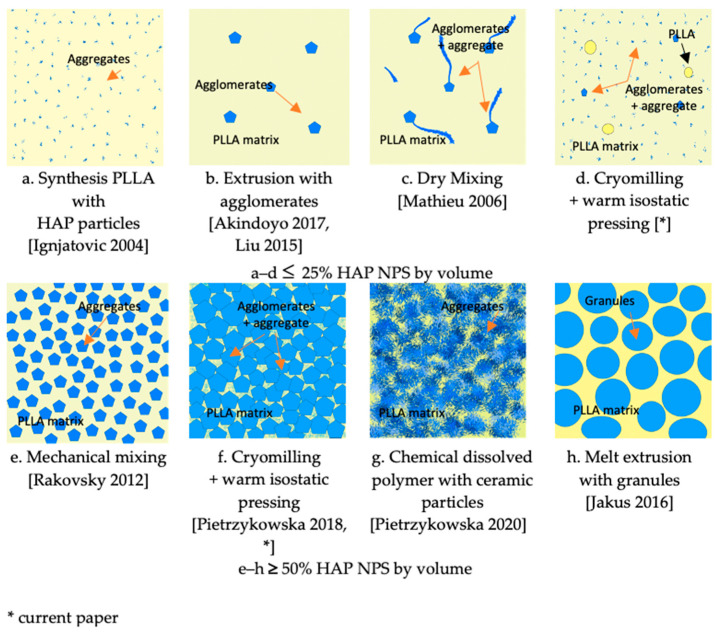
Schematic structures of composites based on bioresorbable polymers depending on calcium phosphate quantity and method of obtaining.

Particular attention should be drawn to the microstructure of 25com, 50com, and 75com. Figure 10 shows a schematic comparison of the composite structures obtained by the present method and various other methods.

In conventional nanocomposites, materials, such as 25com, are usually obtained by adding a small amount of particles during the synthesis of PLLA, or by dissolving the polymer in chemical reagents or melt extrusions [43,44,45,46,48]. Figure 10a,c illustrate microstructures encountered in the literature for 25com. The composite where the ceramic particles are added at the synthesis stage is the most homogeneous one, but the amount of particles is limited (Figure 10a). Melt extrusion is promising because it is characterized by a high dispersion but the long time of extrusion decreases the crystallinity and molecular weight of the polymer, and it is more difficult to extrude the composite with a higher amount of ceramic particles (Figure 10b) [43,46]. Chemical methods help to obtain a high amount of ceramic particles, but the solvents used in them are toxic. The simplest technique is mechanical mixing, but the composite obtained by this method is heterogeneous (Figure 10c). When comparing our method, attention should be paid to the large fragmentation of PLLA and the breakdown of agglomerates during cryomilling and the infiltration of agglomerates during warm isostatic pressing. These two processes contribute to the unique structure of composites and, thus, their homogeneity. Figure 10d presents the obtained structure of 25com.

As far as the 50com composite is concerned, inspection of the fracture surface (Figure 4d) shows that the HAP NPs are homogeneously distributed within aggregates which are 10 μm in size. 

Literature describes the structures of a composite with 40–50% content of calcium phosphates by volume [46,47,48]. Rakovsky et al. [60] obtained composites characterized by a high polymer dispersion through mechanical milling. According to these authors, the thickness of the polymer layer surrounding the ceramics was only 7 nm, which caused the short degradation time of the composite. Figure 10e presents microstructures encountered in the literature for this group of composites. It is an interesting example of an inverse nanocomposite, where we have an equal proportion of the components and polymer is still the matrix of the composite.

Figure 10g,h illustrate microstructures encountered in the literature for 75com. Homogeneity in the 75com composite is higher than in the case of 25com and 50com, which results from the conditions of milling of this composite. When comparing 75com (Figure 10f) with the fracture described in the paper by Pietrzykowska et al.(Figure 10g), it can be noticed that, regardless of the method of obtaining the composite, the fracture nature is almost the same [54]. 

Regardless of the HAP NPs quantity, a connection between the ceramic particles and the polymer matrix is visible in the composites. This is not obvious since many methods of obtaining composites describe the lack of connection and the need to modify the calcium phosphate surface. In order to achieve a connection between hydroxyapatite and the polymer, surface modifiers are applied. The proportion with the HAP NPs content up to 5% and a high dispersion in the nano-scale is regarded as the most favorable proportion for achieving better mechanical properties [43,44]. In terms of biological properties, though, the content above 20% is advisable [11]. 

The connection of nanohydroxyapatite particles with polylactide results from the chemical activity of the nanoparticle surface. It can be inferred that a temperature of 600 °C, which we selected, is optimal for removing water from the surface of the particles, since water is unfavorable to polylactide processing. The structure and activity of the surface of the nanometric hydroxyapatite was, therefore, preserved. 

The microstructure determines the final properties of the material, among others: mechanical strength, flexibility, thermal properties, or degradation time. Therefore, inverse nanocomposites are interesting because of their unknown properties. 

The inverse nanocomposites obtained have mechanical properties similar to bone tissue, a compressive strength ranging from 90–170 MPa, and a Young’s modulus of 3–12 GPa [1,6]. The composites achieve up to four times higher compressive strength than pure PLLA, ranging from 114 to 99 MPa. As the amount of ceramic particles increases, Young’s modulus increases from 1600 to 7317 MPa. 

The flexural strength of our samples increased with the amount of PLLA (up to 28 MPa), but this is still lower compared to natural bone (110 MPa). This is because polylactide itself is brittle. However, by modifying the polylactide, we would probably be able to increase the flexural strength of the composites. Jakus et al. illustrated in their work (using plasticizers in composites with polycaprolactone and other calcium phosphates) that bending strength and elasticity can be improved [1].

The porosity of these materials, which results in lower mechanical properties, must be taken into account. However, from the perspective of bone tissue regrowth, porosity is required. In comparison to the literature, we ended up with lower mechanical properties. However, Rakovsky et al. obtained very high compressive strengths of up to 400 MPa, with a porosity of less than 10%, by pressing at ultra-high pressures of 2 GPa.

The increase in the contact angle in line with the increase in the HAP NPs content corresponds to the literature research. Wenbo Jiang et al. indicate an improvement in the hydrophilicity of surface in line with the addition of hydroxyapatite. Composites with a HAP content of 40 wt%, achieved a contact angle of approx. 68° [74]. This result is comparable to 25com obtained by us. This confirms the expected change in the hydrophilicity of composites in line with the increase in HAP NPs content. 

DSC results indicate that a higher number of HAP NPs in the composite leads to a lower degree of polymer crystallinity. HAP NPs for 50com and 75com composites definitely decreased the temperatures of Tg, Tc, and Tm. Significant differences are seen in the first heating, where, amongst others, water evaporating from the HAP NPs surface decreases Tg and most probably also decreases the molecular weight of the polymer. The way in which the composites are dried and packed determines how much these changes can be controlled, as nanoparticles have a great ability to absorb moisture and gases on their surface. From the cooling curve in Figure 9, it can be concluded that the HAP NPs hinder crystallization.

Ignjatovic et al. show that the ceramic surface causes rearrangement and crystallization of PLLA during composite preparation. As a result, the melting temperature of PLLA, its enthalpy, and crystallinity are changed. Thermomechanical factors cause the breaking of chains and, therefore, a decrease in PLLA molecular mass during forming. In composites with a high amount of ceramic particles, the crystallization proceeds, according to a mechanism that is different from that for pure PLLA after hot pressing. Different mechanisms of crystallization of PLLA in composites with ceramic particles, which are related to the sizes of the crystals, lead to a different appearance of thermal events on DSC curves, and the character of thermal transitions. The amount of ceramic particles in a composite affects the crystallinity degree, glass transition, and melting temperature of the composites investigated in this work. Under the applied pressure, a strain occurs in PLLA globular molecules, they unfold, and linear forms are generated [56]. The knowledge about the impact of HAP NPs on a bioresorbable polymer is an important step towards the control of degradation of and the programming of long-term stability of inverse nanocomposites made of HAP-PLLA.

The 25com and 50com composites are promising, in particular their mechanical properties. The nanoparticles used for their production are similar to these found in natural bone [70]. Therefore, the structure of 50com may be regarded as similar to natural bone. Therefore, the biological properties, including a contact angle and Young’s modulus of 50com, are more conducive to regeneration. These inverse composites are interesting candidates for medical implants of bone tissue in terms of high nanohydroxyapatite content, purity and level of homogeneity. They could be successfully applied to bone implants as bioresorbable bone fillers, such as arthroscopic screws, wedges, and filling.

The long-term stability of bioresorbable implants over time is represented by their primary limitation. The knowledge about the mechanism of their degradation and the possibilities of modification of HAP-PLLA composites are still heavily researched, and the new technologies of their processing, such as 3D printing or warm isostatic pressing, as developed by us, provide a new outlook on the development of these materials. In particular, nano inverse composites are promising because the literature indicates clearly that a higher content of HAP NPs is necessary for bone tissue overgrowth.

The future challenge is to optimize biocompatibility of the composites and to adjust the degradation rates of the nanocomposites to the rate of new bone growth. It seems that this task is achievable by varying the polymer part of the composites. While sufficient mechanical properties were achieved, long-term stability of bioresorbable implants, adjusted to bone regrowth rate over time, is their primary limitation.

## 5. Conclusions

Inverse nanocomposites composed of biodegradable polylactide (PLLA) and hydroxyapatite nanoparticles (HAP NPs) with a nanoceramic content of up to 75% by volume were developed. They were obtained by CAWIP in two stages: the first one is cryomilling of a mixture of both components to create homogeneous granules, and the second one is warm isostatic pressing at the pressure of 65 MPa and the temperature of 170 °C. The method does not require any chemical modifications of the HAP NPs surface or the polymer to achieve a homogenous mixture and a physical connection between HAP NPs and PLLA. 

It is a challenge to create such a bioresorbable composite for an orthopedic implant with the degradation dynamics and time of bone tissue overgrowth that ensure both biological properties and the stability of mechanical properties.

The composite with 50% HAP NPs content seems optimal as a bone implant material in terms of high nanohydroxyapatite content, purity, and homogeneity. The compressive strength is 99 ± 4 MPa, the bending strength is 19 ± 3 MPa, and the Young’s modulus is 3619 ± 295 MPa. The contact angle is high with the value of 50° and the porosity of the material is 25%. The microstructure of the samples shows a uniform phase distribution. The formation of bridges linking the polymer and ceramic component was observed. Natural bone tissue is composed of 8 wt% water, 22 wt% protein, and 70 wt% mineral”, which gives 50 vol% after converting [70].

A decrease in the crystallinity level in the PLLA phase was observed in all composites (25com, 50com, and 75com), in proportion to the HAP NPs content. The decrease in crystallinity of the PLLA phase was the highest for the 75com composite. 

The developed inverse nanocomposite seems a good candidate for such applications as bioresorbable bone implants, such as arthroscopic screws, wedges, and filling. 

## Figures and Tables

**Table 1:** Physical properties of PLLA, HAP NPs (raw), HAP NPs (heated at 600°) components and 25com, 50com, and 75com composites.

Name		Composite Proportion by vol%		Composite Proportion by wt%		HAP NPs [g]	PLLA [g]	Density [g/cm^3^]	Specific Surface Area [m^2^/g]	Grain Size nm
		HAP NPs	PLLA	HAP NPs	PLLA					
1	PLLA	0	100	-	100%	-	1.26	1.26	0.5	-
2	25com	25	75	45%	55%	0.7	0.9	1.67	25	-
3	50com	50	50	70%	30%	1.5	0.6	2.04	42	-
4	75com	75	25	90%	10%	2.2	0.3	2.57	53	-
5	HAP NPs	100%	0	100%	-	3.0	-	3.01	72	29
6	HAP NPs (raw)	-	-	-	-	-	-	2.86	260	8

**Table 2:** Contact angle of PLLA and HAP NPs samples, and composite samples obtained by two-stage forming.

No.	Name	Contact Angle [°]	Increase in Hydrophilicity
1	PLLA	71	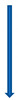
2	25com	59	
3	50com	50	
4	75com	25	
5	HAP NPs	24	

**Table 3:** Changes in mechanical properties of composites, indicating changes in line with increase in HAP NPs in composite.

	Name	Three-Point Bending Test			Compression Test		Porosity [%]
		Young’s Modulus E [MPa]	Ultimate Strength [MPa]	Ultimate Strain [%]	Ultimate Strength [MPa]	Ultimate Strain [%]	
1	PLLA	1603 ± 175	61 ± 6	4.01 ± 0.43	58 ± 8	5.63 ± 0.29	2
2	25com	3289 ± 497	28 ± 6	0.90 ± 0.14	114 ± 2	5.12 ± 0.35	5
3	50com	3619 ± 295	19 ± 3	0.57 ± 0.13	99 ± 4	3.15 ± 0.66	25
4	75com	7317 ± 197	14 ± 2	0.19 ± 0.01	78 ± 7	2.86 ± 0.47	18
5	HAP NPs	8104 ± 38	11 ± 3	0.12 ± 0.02	24 ± 4	1.15 ± 0.16	35

**Table 4:** Thermal properties of composites in line with the increase in HAP NPs content and for PLLA samples. Tg: glass transition temperature; Tm: melting temperature; Tc: cold crystallization peak; ΔCp: specific heat capacity; Δ*Hm*: melting enthalpy; Xc: crystallinity.

Process /Parameter	PLLA		25com		50com		75com	
	Heating 1	Heating 2	Heating 1	Heating 2	Heating 1	Heating 2	Heating 1	Heating 2
Tg (C°)	57.50	60.70	51.50	**61.70**	50.50	58.90	52.0	60.4
Tg _Heating 2- 1_	↑3.2		↑10.2		↑8.4		↑8.4	
Tc (C°)	-	110.70 163.9	-	107.90 163.5	-	104.08 163.1	-	108.50 163.2
Tm (C°)	197.4	180.3	197.70	181.70	194.00	179.40	194.50	180.00
Tm _Heating 1-2_	17.1		↓16.4		↓14.6		↓14.5	
Δ*Hm*		45.28		21.26		9.76		2.8
X_c_ (%)	91.4	26.5	67.8	9.5	49.8	7.9	29.8	3.0

## Data Availability

The data presented in this study are available on request from the corresponding author.
